# Reduced Regional NREM Sleep Slow-Wave Activity Is Associated With Cognitive Impairment in Parkinson Disease

**DOI:** 10.3389/fneur.2021.618101

**Published:** 2021-02-19

**Authors:** Simon J. Schreiner, Lukas L. Imbach, Philipp O. Valko, Angelina Maric, Rina Maqkaj, Esther Werth, Christian R. Baumann, Heide Baumann-Vogel

**Affiliations:** ^1^Department of Neurology, University Hospital Zurich, University of Zurich, Zurich, Switzerland; ^2^Clinical Neuroscience Center, University Hospital Zurich, University of Zurich, Zurich, Switzerland; ^3^Sleep & Health Zurich, University of Zurich, Zurich, Switzerland

**Keywords:** Parkinson disease, sleep, slow-wave sleep, cognition, cognitive impairment, polysomnography, EEG, spectral analysis

## Abstract

Growing evidence implicates a distinct role of disturbed slow-wave sleep in neurodegenerative diseases. Reduced non-rapid eye movement (NREM) sleep slow-wave activity (SWA), a marker of slow-wave sleep intensity, has been linked with age-related cognitive impairment and Alzheimer disease pathology. However, it remains debated if SWA is associated with cognition in Parkinson disease (PD). Here, we investigated the relationship of regional SWA with cognitive performance in PD. In the present study, 140 non-demented PD patients underwent polysomnography and were administered the Montréal Cognitive Assessment (MoCA) to screen for cognitive impairment. We performed spectral analysis of frontal, central, and occipital sleep electroencephalography (EEG) derivations to measure SWA, and spectral power in other frequency bands, which we compared to cognition using linear mixed models. We found that worse MoCA performance was associated with reduced 1–4 Hz SWA in a region-dependent manner (*F*_2, 687_ =11.67, *p* < 0.001). This effect was driven by reduced regional SWA in the lower delta frequencies, with a strong association of worse MoCA performance with reduced 1–2 Hz SWA (*F*_2, 687_ =18.0, *p* < 0.001). The association of MoCA with 1–2 Hz SWA (and 1–4 Hz SWA) followed an antero-posterior gradient, with strongest, weaker, and absent associations over frontal (rho = 0.33, *p* < 0.001), central (rho = 0.28, *p* < 0.001), and occipital derivations, respectively. Our study shows that cognitive impairment in PD is associated with reduced NREM sleep SWA, predominantly in lower delta frequencies (1–2 Hz) and over frontal regions. This finding suggests a potential role of reduced frontal slow-wave sleep intensity in cognitive impairment in PD.

## Introduction

Parkinson disease (PD) is the second most common neurodegenerative disorder after Alzheimer disease (AD). The majority of PD patients will develop progressive cognitive impairment, likely due to ongoing neurodegeneration with Lewy body and concomitant AD pathology (1). As prevention and treatment of cognitive impairment remain major challenges, we need to identify modifiable risk factors.

Slow-wave sleep (SWS), i.e., deep non-rapid eye movement (NREM) sleep, supports cognition and could modulate neurodegeneration by influencing pathological proteins such as alpha-synuclein, tau, and beta-amyloid (2–4). Sleep induction decreases whereas sleep restriction increases pathological proteins in rodent brain tissue and interstitial fluid and human cerebrospinal fluid (2–4). These dynamics may specifically depend on SWS, as SWS disruption—but not partial sleep restriction with preserved SWS—modulates beta-amyloid levels in humans (5, 6). Slow-wave activity (SWA) quantifies sleep slow waves, the electroencephalographic hallmark of SWS. SWA likely indicates how strongly SWS could modulate neurodegeneration, as SWA correlates positively with glymphatic clearance in mice (7), and negatively with AD-related cognitive impairment and pathology in humans (8–11).

In PD, progressive deterioration of SWS has been described, with altered slow wave morphology at prodromal stages (12), reduced SWA in *de novo* patients (13), and reduced SWS amount in advanced stages (14). Moreover, impaired SWA has been linked with motor complications and progression (15, 16).

However, the relationship of SWA with cognition in PD remains unclear, as findings on this topic are inconsistent. A detailed sleep EEG analysis reported no association of SWA with cognitive impairment in PD patients, but instead highlighted the importance of other sleep EEG alterations over posterior derivations (17). In contrast, a recent study linked SWA, a priori selected from frontal derivations, to global cognition, but the sample included only 32 PD patients (18). Therefore, the aim of our study was to clarify the relationship of SWA with cognition in PD by analyzing SWA and its regional variation in a larger sample of non-demented PD patients. In addition, we tested the hypothesis that the relationship between cognition and SWA in PD is regional, preferentially involving frontal regions, which have been implicated with slow wave generation and altered SWA related to aging and AD pathology (9–11, 19, 20).

## Method

This study was conducted after ethical approval (Kantonale Ethikkommission Zürich) and aligned with the Declaration of Helsinki. We included all consecutive PD patients, who had undergone a strictly predefined diagnostic work-up within the highly specialized medicine program to evaluate eligibility for deep brain stimulation between January 2012 and July 2018. This standardized work-up included cognitive testing and polysomnography, irrespective of cognitive or sleep complaints, and results were thoroughly documented. From this documentation, we extracted demographic and clinical data, including Hoehn and Yahr stage, Unified Parkinson's Disease Rating Scale motor part (after >12 h without PD medication), motor phenotype (21), and medication at the timing of polysomnography, including the levodopa equivalent dose (LED) (22), and sleep medications, i.e., drugs that could affect sleep (antidepressants, antipsychotics, acetylcholine-esterase-inhibitors, benzodiazepines, benzodiazepine-receptor-agonists, stimulants, melatonin), which we further analyzed if taken by more than one patient.

Trained neuropsychologists or neurologists administered the Montréal Cognitive Assessment (MoCA) during medical “on” (23). For descriptive purposes, we screened patients with MoCA ≥ 26 as cognitively normal, and patients with MoCA <26 as cognitively impaired, because this MoCA cutoff has been shown to have a high diagnostic accuracy to screen for mild cognitive impairment in PD (24). Inclusion criteria were diagnosed PD (25), and available MoCA and polysomnography. Exclusion criteria were other forms of Parkinsonism, a clinical diagnosis of dementia (26), or incompatible polysomnography (e.g., no sleep).

Patients underwent a single-night video-polysomnography in the in-house sleep laboratory (Embla N7000, RemLogic v3.2). Experienced sleep experts (E. W., H. B. V., S. J. S.) manually scored sleep stages according to the AASM manual (27). The following variables were derived: total sleep time, sleep efficiency, percentages of wake after sleep onset, rapid eye movement (REM) and non-REM (NREM) sleep stages (N1, N2, N3), sleep latency (time until N2), indices (events/hour) for apnea-hypopnea, arousal, and periodic limb movements during sleep, and REM sleep behavior disorder (RBD) according to international criteria (28).

To test our hypothesis that cognitive impairment is associated with SWA, predominantly over frontal regions, we performed spectral analysis from six EEG derivations covering frontal, central, and occipital regions (F3, F4, C3, C4, O1, O2, all referenced to contralateral mastoid). Preprocessing of EEG and spectral analysis were performed using previously described routines (0.5 Hz high-pass and 40 Hz low-pass filtering, visual inspection and semi-automatic artifact removal; Fast Fourier Transformation; 5 s-epochs) (29). We documented the number of artifact-free EEG epochs from each EEG derivation, to exclude possible confounding. On artifact-free EEG, aligned with manual sleep scoring, we averaged spectral power during N2 and N3 over the entire night in the slow oscillation (0.5–1 Hz), delta (1–4 Hz), theta (4–8 Hz), alpha (8–13 Hz), and beta (13–20 Hz) frequency bands. Additional SWA bins were computed (1–2, 2–3, 3–4 Hz) because previously reported associations of AD-related cognitive impairment and pathology with SWA where particularly evident in the lower delta frequencies <2 Hz (9–11). We normalized band power to total 0.5–40 Hz power during N2 and N3, to avoid bias due to interindividual differences in EEG amplitude and capture the relative contributions of the 1-Hz segmented SWA to NREM power. For improved interpretation, we also report on absolute NREM sleep power densities.

The statistical analysis was carried out with SPSS (v23.0; IBM, Armonk, NY). We studied the relationship of cognition with regional NREM sleep EEG power within a linear mixed model framework. For each frequency band, separate linear mixed models were calculated, with NREM sleep EEG power as dependent variable, and hemisphere (left, right), region (frontal, central, occipital), MoCA performance, and the interactions of MoCA with hemisphere and region as fixed effects. A random effect was defined for patient intercept. All models were controlled for age, sex, disease duration, years of education, motor phenotype (akinetic-rigid yes/no), LED, and the sum of sleep medications. We used estimates of fixed effects for significant interactions of MoCA with region or hemisphere, to locate regional effects. All continuous variables were transformed into z scores, to allow comparisons among estimates. Skewed variables were log-transformed. We assessed normality of variables and residuals by applying the Kolmogorov-Smirnov test and plotting. To correct for multiple testing, the Bonferroni-Holm method was applied. For descriptive purposes, we also compared clinical and polysomnography data and NREM sleep EEG power densities with significant interaction effects between patients with and without cognitive impairment, using the Chi^2^ test for categorical data and—depending on normal distribution—the independent sample *t*-test or Wilcoxon rank-sum test for continuous data. Bivariate correlations were used to illustrate the strongest association of MoCA with NREM sleep EEG power. We consider corrected *p*-values < 0.05 as significant but report uncorrected *p*-values whenever informative.

## Results

Tables 1, 2 summarize clinical and polysomnography characteristics. We excluded three patients due to incompatible polysomnography (no N2 and N3) and seven derivations with poor quality on one hemisphere.

**Table 1:** Clinical characteristics.

	**All PD patients (*n* = 140)**	**Cognitively normal (*n* = 105)**	**Cognitively impaired (*n* = 35)**	***p*^**†**^**
Age (years)	62.6 ± 9.1	62.0 ± 8.8	64.5 ± 9.7	0.09
Females, *n* (%)	62.0 (44.3)	48.0 (45.7)	14.0 (40.0)	0.56
Disease duration (years)	10.4 ± 5.2	10.1 ± 5.0	11.1 ± 5.6	0.34
Hoehn and Yahr stage	2.3 ± 0.6	2.3 ± 0.6	2.4 ± 0.5	0.36
Montréal Cognitive Assessment	26.9 ± 2.6	28.2 ± 1.4	23.1 ± 1.6	n.a.
Education (years)	12.9 ± 3.3	13.2 ± 3.3	11.9 ± 3.2	0.03
UPDRS III	37.6 ± 13.3	37.0 ± 13.1	39.2 ± 14.0	0.49
Akinetic rigid subtype, *n* (%)	62.0 (44.3)	44.0 (41.9)	18.0 (51.4)	0.33
LED total (mg/d)	1,009.6 ± 444.0	1,038.6 ± 475.0	922.8 ± 325.0	0.22
LED dopamine agonists (mg/d)	144.6 ± 137.1	155.0 ± 141.7	113.7 ± 118.6	0.16
LED levodopa (mg/d)	799.4 ± 418.8	815.3 ± 443.1	752.0 ± 336.5	0.64
Antidepressants, any, *n* (%)	44 (31.4)	28 (26.7)	16 (45.7)	0.04
Antidepressants, sedating^‡^, *n* (%)	28 (20.0)	15 (14.3)	13 (37.1)	<0.001
Antidepressants, activating^§^, *n* (%)	18 (12.9)	14 (13.3)	4 (11.4)	0.77
Acetylcholine-esterase-inhibitors, *n* (%)	4 (2.9)	1 (1)	3 (8.6)	0.02
Antipsychotics, *n* (%)	7 (5.0)	3 (2.9)	4 (11.4)	0.044
Benzodiazepines, any, *n* (%)	18 (12.9)	11 (10.5)	7 (20.0)	0.16
Clonazepam, *n* (%)	8 (5.7)	6 (5.7)	2 (5.7)	0.99
Other benzodiazepines, *n* (%)	10 (7.1)	5 (4.8)	5 (14.3)	0.06
Benzodiazepine receptor agonists, *n* (%)	8 (5.7)	8 (7.6)	0 (0.0)	0.93

Means ± standard deviations are shown; Comparison of PD patients with and without cognitive impairment; uncorrected p-values are displayed;

§ Selective serotonin reuptake inhibitors and selective serotonin and norepinephrine reuptake inhibitors;

‡ *Trazodone, mirtazapine, tricyclic antidepressants. PD, Parkinson disease; UPDRS III, part III (motor part) of the Unified Parkinson's Disease Rating Scale; LED, levodopa-equivalent dose*.

**Table 2:** Frontal NREM sleep EEG power and polysomnography.

	**All patients (*n* = 140)**	**Cognitively normal (*n* = 105)**	**Cognitively impaired (*n* = 35)**	***p*^**†**^**
0.5–1 Hz slow oscillation (%)	49.3, 8.9	49.23, 8.9	49.5, 9.1	0.91
1–4 Hz SWA (%)	37.8, 5.7	38.5, 5.3	36.2, 662	0.02
1–2 Hz SWA (%)	26.5, 4.7	27.1, 4.5	24.8, 8.4	0.01
2–3 Hz SWA (%)	9.6, 1.9	9.6, 1.7	9.3, 2.3	0.26
3–4 Hz SWA (%)	5.2, 1.2	5.1, 1.0	5.3, 1.4	0.54
4–8 Hz theta (%)	10.8, 3.6	10.5, 3.6	11.4, 3.6	0.20
8–13 Hz alpha (%)	5.9, 3.0	5.2, 2.9	6.7, 3.5	0.49
13–20 Hz beta (%)	2.2, 1.4	2.2, 0.9	2.4, 1.4	0.93
Sleep period time (min)	422.0, 50.6	418.4, 53.2	432.8, 40.5	0.18
Total sleep time (min)	325.0, 76.0	325.6, 77.2	323.3, 73.6	0.70
Wake After Sleep Onset (min)	96.9, 60.1	92.8, 59.1	109.5, 62.2	0.16
Sleep efficiency (%)	74.6, 15.3	75.3, 15.6	72.5, 14.6	0.23
Sleep latency to N2 (min)	25.1, 40.2	23.9, 41.0	28.7, 38.1	0.08
N1 (%)	14.6, 9.8	14.2, 8.5	15.9, 12.9	0.64
N2 (%)	38.6, 13.8	39.6, 13.2	35.4, 15.2	0.13
N3 (%)	13.2, 8.9	13.1, 8.9	13.3, 8.9	0.87
R (%)	11.5, 6.7	11.8, 6.7	10.6, 6.5	0.35
Arousal index (events/hour)	12.7, 8.5	12.7, 9.0	12.6, 7.0	0.56
PLMS (events/hour)	9.4, 20.8	8.1, 18.1	13.0, 27.0	0.41
Apnea-hypopnea index (events/hour)	5.2, 7.8	5.4, 8.1	4.3, 6.7	0.58
REM sleep behavior disorder, *n* (%)	110 (78.6)	81 (77.1)	29 (82.9)	0.48

Means ± standard deviations are shown;

† *Comparison of PD patients with and without cognitive impairment; uncorrected p-values are displayed. Sleep stages are presented as percentages of sleep period time. Spectral power densities are presented as percentages of total power during NREM sleep and averaged across hemispheres from frontal derivations (F3, F4). PD, Parkinson disease; SWA, slow-wave activity; N1–3, non-rapid eye movement sleep stages 1–3; R, rapid eye movement sleep; PLMS, periodic limb movements during sleep*.

Cognitive impairment as indicated by worse MoCA performance was associated with reduced regional 1–4 Hz SWA (MoCA^*^region: *F*_2, 687_ = 11.67, *p*-corrected < 0.001). This effect was driven by a reduction in the lower delta frequencies, as indicated by a strong association of worse MoCA performance with reduced regional 1–2 Hz SWA (MoCA^*^region interaction: *F*_2, 687_ =18.0, *p*-corrected < 0.001) but not with SWA in higher delta frequencies (Table 3). Further, worse MoCA performance was weakly associated with increased regional alpha power. An association of worse MoCA performance with increased regional theta power did not survive correction for multiple testing (Table 3). Main effects of MoCA were not significant (only a trend was observed for 1–2 Hz SWA: *F*_2, 687_ = 3.7, *p*-*un*corrected = 0.05), indicating that cognition was associated with NREM sleep EEG power in a region-dependent manner.

**Table 3:** Effects of cognitive performance (MoCA) on regional NREM sleep EEG power.

**Dependent variable**	***F*_**2, 687**_**	***p***
0.5–1 Hz slow oscillation	0.15	0.86
1–4 Hz SWA	11.67	^*^ <0.0001
1–2 Hz SWA	18.00	^*^ <0.00001
2–3 Hz SWA	1.14	0.32
3–4 Hz SWA	0.90	0.43
4–8 Hz theta	3.32	0.04
8–13 Hz alpha	5.67	^*^0.03
13–20 Hz beta	0.49	0.61
**Parameter**	**Estimates (95% CI)**	
Frontal 1–4 Hz SWA	0.21 (0.12; 0.29)	<0.000001
Central 1–4 Hz SWA	0.16 (0.07; 0.25)	<0.001
Frontal 1–2 Hz SWA	0.24 (0.16; 0.33)	<0.0000001
Central 1–2 Hz SWA	0.19 (0.11; 0.28)	<0.0001
Frontal 4–8 Hz theta	−0.10 (−0.18; −0.02)	0.02
Central 4–8 Hz theta	−0.06 (−0.14; 0.02)	0.12
Frontal 8–13 Hz alpha	−0.10 (−0.15; −0.04)	<0.001
Central 8–13 Hz alpha	−0.06 (−0.12; −0.01)	0.03

The upper part of the table shows all fixed effects for the MoCA*region interaction term in the linear mixed model analysis. The lower part of the table shows estimates of significant fixed effects for the interaction of MoCA and region. The occipital region was not significant and is used for comparison in this contrast.

* *p-value after correction for multiple testing. CI, 95% confidence intervall; MoCA, Montréal Cognitive Assessment*.

**Figure 1:**
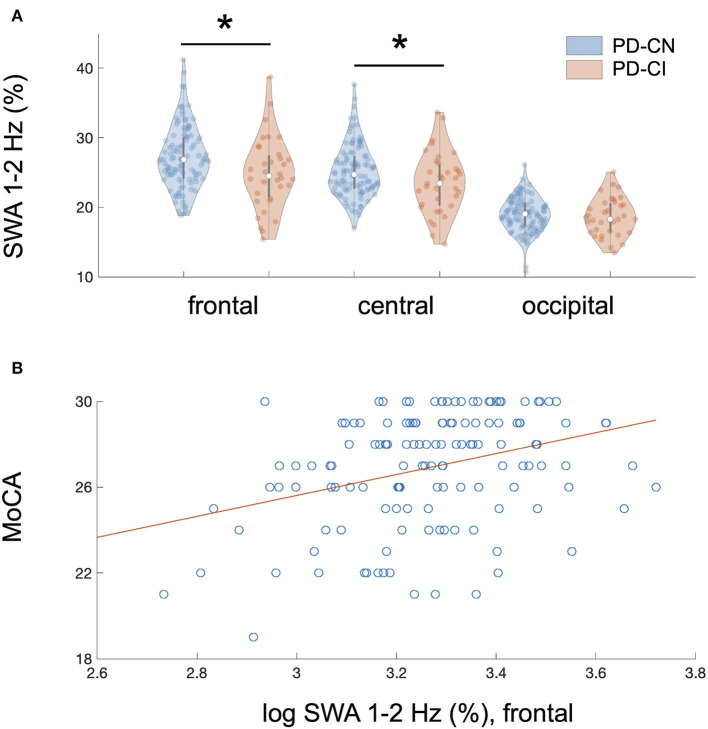
Regional association of reduced NREM sleep SWA with cognitive impairment in PD patients. **(A)** Cognitive impairment showed a regional and frequency-specific association with reduced NREM sleep SWA, predominantly in the lower delta frequencies (1–2 Hz), in 140 non-demented patients with Parkinson disease (PD); linear mixed model analysis: MoCA*region interaction, *F*_2, 687_ = 18.0, *p* < 0.00001 after correction for multiple testing and controlling for relevant covariates. The association of MoCA performance with SWA followed an antero-posterior gradient with strongest, weaker, and absent associations over frontal, central, and occipital derivations, respectively. Plot A represents a binary visual representation of regional 1–2 Hz SWA across cognitively impaired (*n* = 35, labeled as PD-CI, red color) and cognitively normal PD patients (*n* = 105, labeled as PD-CN, blue color). **(B)** Cognitive impairment and regional NREM sleep 1–2 Hz SWA showed the strongest association over frontal EEG derivations. This relationship is illustrated for averaged bifrontal 1–2 Hz SWA in all 140 PD patients; bivariate correlation: rho = 0.33, *p* < 0.0001. The Montréal Cognitive Assessment (MoCA) was used to screen for cognitive impairment. Slow-wave activity (SWA) was normalized to total spectral power during NREM sleep (0.5–40 Hz) and log-transformed, yet image A displays raw data.*significant after controlling for multiple testing.

The association of MoCA performance with SWA followed an antero-posterior gradient, with strongest, weaker, and absent associations over frontal, central, and occipital derivations, respectively (Table 3). Figure 1A provides a binary visual representation of this relationship across patients with and without cognitive impairment. Figure 1B displays a continuous visual representation of the strongest effect, i.e., the association of worse MoCA performance with reduced frontal 1–2 Hz SWA (frontal: rho = 0.33, *p* < 0.001; central: rho = 0.28, *p* < 0.001). Similar but weaker findings were obtained when absolute instead of relative NREM sleep power was used (results not shown).

We did not observe any associations of MoCA with spectral power in relation to hemisphere (no significant MoCA^*^hemisphere interaction), suggesting that the relationship of MoCA with NREM sleep EEG did not depend on hemispheric differences. Thus, averaged regional NREM sleep power densities across hemispheres were used in further analyses. We observed an additional significant fixed effect of sleep medications, which related to reduced 1–4 Hz SWA (*F*_1, 132_ = 9.8, *p*-corrected = 0.02) and 1–2 Hz SWA (*F*_1, 132_ = 8.5, *p*-corrected = 0.03).

Out of the 140 patients, 35 (25%) screened positive and 105 (75%) negative for cognitive impairment. Conventional polysomnography characteristics did not differ between patients with and without cognitive impairment. Compared to cognitively normal patients, patients with cognitive impairment more frequently took sedating antidepressants, acetylcholine-esterase-inhibitors, antipsychotics, and (at trend-level significance) other benzodiazepines, and showed fewer education years (see Table 1).

## Discussion

In 140 non-demented PD patients, we investigated whether cognitive impairment is associated with SWA, i.e., objectively measured SWS intensity. We found that worse MoCA performance was associated with reduced SWA, particularly in the lower delta frequencies (1–2 Hz) and over frontal EEG derivations. This finding adds evidence that reduced SWA is associated with cognitive impairment in PD. Moreover, the association of SWA with cognition was regional and frequency-specific, which resembles previously reported associations of frontal SWA—especially low-frequency SWA—with cognitive impairment and pathology in a context of early AD (8–11, 20). Thus, reduced SWA, predominantly over frontal derivations, may represent an overlapping marker of early cognitive impairment in neurodegenerative diseases such as PD and AD. However, it remains unclear whether neurodegeneration impacts SWS, or vice versa, or both.

In PD, shared etiologies could cause sleep and cognitive impairments, as both show overlapping patho-anatomical correlates, with alpha-synuclein and tau accumulation in brain regions regulating cognition and sleep, including brainstem, hypothalamic and limbic areas (30). Moreover, several candidate mechanisms underlying cognitive impairment in PD overlap with those linking disturbed sleep with neurodegeneration, including glial alterations, oxidative stress, or pathological protein accumulation (1, 31, 32).

Although not a primary outcome of this study, we did not observe differences in polysomnographic parameters other than reduced SWA when comparing cognitively impaired with cognitively normal patients. In contrast, previous studies linked cognitive impairment in PD with various sleep abnormalities, including reduced sleep efficiency, sleep apnea, RBD, REM sleep slowing, or reduced spindles (17, 33–38). However, these studies are not directly comparable due to differences in methods and patient characteristics. For example, some previous studies used actigraphy, assessed cognitive subdomains (33, 34), or included patients with higher degrees of sleep and cognitive impairments (36, 37). Although our study is cross-sectional, our finding on reduced NREM SWA resonates with longitudinal studies linking cognitive decline in PD with other NREM sleep abnormalities such as reduced spindles or SWS amount (17, 38, 39). Importantly, compared to SWS amount, SWA may be more sensitive to neurodegenerative cognitive impairment, as previously discussed (8).

We are aware of two previous studies that compared SWA to cognition in PD, with inconsistent findings. While a detailed sleep EEG analysis in 68 PD patients reported no association of cognitive impairment or decline with SWA (NREM delta power) (17), a recent study linked frontal SWA to global cognition in 32 PD patients (18). Compared to these studies, our analysis includes a larger sample (*n* = 140) of non-demented PD patients and 1-Hz- segmentation of regional SWA. Thus, our data adds evidence that SWA is linked to cognition in PD. In addition, our findings suggest that the relationship of cognition and SWA in PD is regional and frequency-specific. The pattern of specific involvement of frontal regions and predominant alteration of lower delta frequencies is consistent with a physiological antero-posterior gradient of SWA <2 Hz (40), local slow wave generation (19) and altered SWA related to aging and AD pathology (9–11, 20). Thus, a possible explanation for the discrepant findings on SWA and cognition are the distinct cognitive phenotypes and related trajectories in PD (41, 42). Reduced frontal SWA could be a possible marker of frontal-executive (18) rather than posterior cognitive dysfunction and incipient dementia in PD (17).

Our finding should be interpreted with caution given several limitations. We only cross-sectionally screened for cognitive impairment, without assessing cognitive self-reports, subdomains or longitudinal trajectories. On the other hand, the fact that simple cognitive screening revealed our finding might indicate that the link between SWS and cognition in PD is rather strong. Lack of a habituation polysomnography introduces a “first night effect” as possible bias. The retrospective study design is a limitation, but all procedures and documentations followed strictly predefined routines as part of a diagnostic work-up prior to deep brain stimulation. Thus, patient selection may limit generalization. On the other hand, our sample is representative for many non-demented PD patients with moderate disease severity, who could benefit from interventions to prevent cognitive decline. We were unable to systematically consider all factors that may influence cognition and sleep, such as depression, nocturia, or nocturnal “off” symptoms. Patients continued medication during polysomnography, and cognitively impaired patients more frequently took several medications with possible effects on sleep and cognition. Although this may simply reflect pharmacological management in PD patients with more impaired cognition and sleep, we also observed that these medications were additionally associated with less SWA. Importantly, though, accounting for these pharmacological effects did not attenuate the association of MoCA performance with SWA. Future studies may investigate pharmacological influences on SWS and cognition in more depth.

Taken together, the present study suggests that cognitive impairment in PD patients is associated with reduced SWA, particularly in the lower delta frequencies (1–2 Hz) and in frontal regions. Our study adds evidence that SWS could play a beneficial role in PD.

Future research should relate SWS characteristics to longitudinal trajectories, cognitive sub-domains in larger samples, and neuroimaging or fluid biomarkers, and explore the potential of SWS as a biomarker and therapeutic target in PD and related disorders.

## Data Availability Statement

The raw data supporting the conclusions of this article will be made available by the authors, without undue reservation.

## Ethics Statement

The study was reviewed and approved by the local ethics committee (Kantonale Ethikkommission Zürich, Switzerland). The patients provided written informed consent for further use of health-related data.

## Author Contributions

SS: conceptualization, data acquisition, data analysis, data visualization, writing of the draft, and funding acquisition. LI and PV: data analysis, review and editing of the draft. AM: conceptualization, review and editing of the draft. RM: data acquisition, review and editing of the draft. EW: data analysis, resources, review and editing of the draft. CB: conceptualization, review and editing of the draft, funding acquisition, supervision, and resources. HB-V: conceptualization, data acquisition, review and editing of the draft, and supervision. All authors contributed to the article and approved the submitted version.

## Conflict of Interest

The authors declare that the research was conducted in the absence of any commercial or financial relationships that could be construed as a potential conflict of interest.
